# Empowering the discovery of novel target-disease associations via machine learning approaches in the open targets platform

**DOI:** 10.1186/s12859-022-04753-4

**Published:** 2022-06-16

**Authors:** Yingnan Han, Katherine Klinger, Deepak K. Rajpal, Cheng Zhu, Erin Teeple

**Affiliations:** Translational Sciences, Sanofi US, Framingham, MA 01701 USA

**Keywords:** Open targets, Drug discovery, Machine learning, XGBoost, Target indication expansion, Drug repurposing, Data Integration, Feature engineering

## Abstract

**Background:**

The Open Targets (OT) Platform integrates a wide range of data sources on target-disease associations to facilitate identification of potential therapeutic drug targets to treat human diseases. However, due to the complexity that targets are usually functionally pleiotropic and efficacious for multiple indications, challenges in identifying novel target to indication associations remain. Specifically, persistent need exists for new methods for integration of novel target-disease association evidence and biological knowledge bases via advanced computational methods. These offer promise for increasing power for identification of the most promising target-disease pairs for therapeutic development. Here we introduce a novel approach by integrating additional target-disease features with machine learning models to further uncover druggable disease to target indications.

**Results:**

We derived novel target-disease associations as supplemental features to OT platform-based associations using three data sources: (1) target tissue specificity from GTEx expression profiles; (2) target semantic similarities based on gene ontology; and (3) functional interactions among targets by embedding them from protein–protein interaction (PPI) networks. Machine learning models were applied to evaluate feature importance and performance benchmarks for predicting targets with known drug indications. The evaluation results show the newly integrated features demonstrate higher importance than current features in OT. In addition, these also show superior performance over association benchmarks and may support discovery of novel therapeutic indications for highly pursued targets.

**Conclusion:**

Our newly generated features can be used to represent additional underlying biological relatedness among targets and diseases to further empower improved performance for predicting novel indications for drug targets through advanced machine learning models. The proposed methodology enables a powerful new approach for systematic evaluation of drug targets with novel indications.

**Supplementary Information:**

The online version contains supplementary material available at 10.1186/s12859-022-04753-4.

## Background

The Open Targets (OT) platform (https://www.targetvalidation.org/) originated in 2014 as the Center for Therapeutic Target Validation (CTTV). The platform is a public–private research partnership aimed at integration of multiple target-disease linkage evidences and supporting the development of exploratory methods to facilitate drug target selection and validation [1]. A broad range of target-disease association features are aggregated in OT from public domain information sources, specifically genetic association, somatic mutation, pathway biology, transcriptomics, text mining, animal model, and known drug status. Summarized association features in Open Targets are reported for target-disease pairs as computed association scores for these relationships [2]. As the accumulation of data continues, the Open Targets platform has consolidated evidence for over 11 million potential target-disease pairs in the latest 21.04 release (https://blog.opentargets.org/next-gen-platform-released/). The consolidated target-disease association pairs have the potential to be usefully explored via data mining and algorithmic methods. OT currently serves as an important resource for academic and pharmaceutical industry scientists for generating hypotheses on new target-disease relationships as well as for drug repurposing and target indication expansion, among other activities [3].

There exist a number of studies that have already reported the use of OT data for such applications. For instance, Freudenberg et al*.* introduced a systematic approach for utilizing Open Targets platform data to prioritize potential new disease indications, specifically for G-protein coupled receptors and their ligands. Targeting of GPR35 for inflammatory bowel disease and CXCR4 for viral infection were illustrated as interesting hypotheses derived from this approach [4]. In another study by Khaladkar et al*.* [5], the authors proposed a workflow which computes target-disease score thresholds to mine Open Targets platform data for repositioning opportunity identification, in which vitiligo was identified as a potential indication for target melanocortin 1 receptor (MC1R). Shaher et al*.* performed a comprehensive review on OT data to prioritize and identify the novel targets associated with diabetic cardiomyopathy. Several targets have been identified by narrowing down the OT evidence with exclusion criteria [6]. The discoveries from these methods were mostly based on setting certain filtering criteria. Meanwhile there were also pioneer studies that have shown the potential power of data-driven approaches using machine learning models on OT data for drug-target identification tasks, where the models can learn patterns of disease to target associations without prior biological dependency information. Ferrero et al*.* [7] constructed four machine learning models to predict whether a gene is a drug target or non-target using five OT data types (genetic association, somatic mutation, pathway biology, RNA-expression, animal model) as input features. Their work assessed feature importance for target identification and showed that gene-disease linkage evidence was sufficient to predict novel therapeutic targets effectively, confirming that those types of evidence were essential. However, machine learning approaches in their work were mainly focused on predicting whether a gene is a drug target or non-target, while the task of predicting drug targets and their novel indications has not yet been fully explored.

**Fig. 1:**
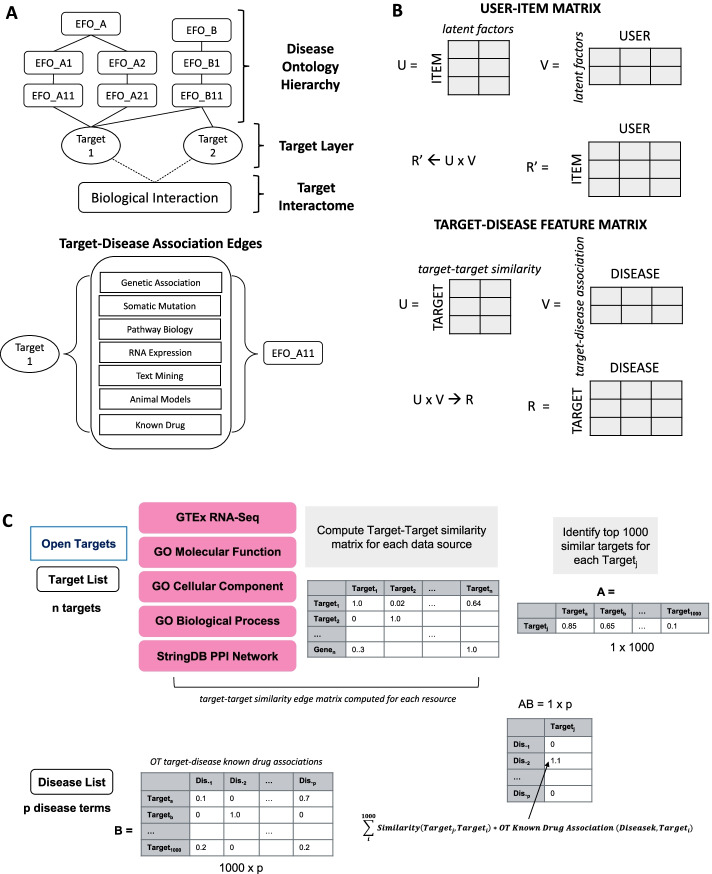
Overview of Open Targets data and generation of newly computed features. Open Targets association evidence network edge weights are annotated for evidence from multiple sources (**a**). Novel target-disease association features generated from target-target similarity and target-disease matrices compared with factors used in calculation of a user-item matrix (**b**). Target-disease arrays are generated for each information source and association evidence for known drug status (**c**)

Novel target-disease indication searches present unique and challenging problems for computational scientists. Despite the vast number of target-disease association pairings already integrated on the Open Target platform, many drug targets are functionally pleiotropic and thus might be good targets for multiple indications or might have associations diseases which are not compatible with compounds under study. This creates challenges when using Open Targets to generate strong target-indication hypotheses due to the limited number of features on these pairs available to distinguish among them. For example, while genetic association evidence among target-disease pairs reflects the current state of scientific knowledge of their relatedness, the platform does not include aggregated evidence on target tissue-specific biological relatedness. Lacking this information, searches overlook the fact that genes co-expressed in the same tissues may share higher-level processes that functionally connect them [8], and tissue-specific co-expression patterns could even contain alternative targets for a disease. Another limitation of OT is the lack of interconnectivities among targets, which would be expected to reflect relevant and disease-implicated biological processes. Targets which participate together in disease-relevant pathways may not share a disease term if only one of these targets has been studied relative to the disease of interest, however, these relationships would become more discoverable if biological interconnectedness could be further linked in an information network model (Fig. 1a). Such target interconnectivities have been considered as potentially capturing evidence of some not-yet-known relationships [9]. Additionally, the semantic similarity between Gene Ontology (GO) terms is an essential step in bioinformatics research to study gene functional similarities [10]. This functional similarity is very informative for drug repurposing as genes with high functional similarities have a greater probability of being implicated in a same phenotype or disease [11]. However, integration of such data sources is not yet available through the Open Target platform.

To date, these limitations have restricted OT target-disease discovery performance and motivate us to develop and evaluate a novel approach, which starts with the integration OT association information with additional target-disease biological relatedness features which are then evaluated for prediction of target-disease therapeutic status by machine learning model training and prediction. We hypothesized that the integration of newly generated features would further strengthen the representation of underlying biological relatedness among targets and diseases, and that inclusion of these novel features could boost power and accuracy for the identification and prioritization of target-indications pairs for drug discovery.

Here, we present the key steps in our approach. First, we generated new target-disease association features as a product of a known target-indication matrix and target-target similarity matrices derived from our orthogonal information resources. Our proposed method represents a novel drug discovery-specific adaptation of collaborative filtering [12] in recommender systems, where the user-item matrix is inferred as the product of item-factor and user-factor matrices, with factors learned from experimental data(Fig. 1b). In our study, we explicitly encoded a set of new target-disease relatedness matrices, each of which was computed as the product of a matrix on OT target-disease score with target-target information matrix newly derived from one of the external data sources (Fig. 1c, see Materials and Methods for details). Data sources used to generate the target-target similarity matrices were from the Genotype-Tissue Expression (GTEx) [13]; Gene Ontologies on Molecular Function (MF), Cellular Component (CC) and Biological Process (BP) annotations [14, 15]; Functional interactions among targets which were represented by embedding them into a protein–protein interaction (PPI) network from STRING database (version11) [16] using the Node2Vec algorithm [17].

We then evaluated how these newly generated features might perform for representing underlying biological relatedness among targets and diseases. We trained three machine learning models and evaluated their performance using OT association evidence and newly generated biological relatedness features for the task of predicting whether a given target-disease pair might have a known drug indication, particularly testing whether a subset of targets hidden during feature generation and training could be correctly predicted from their encoded biological relatedness. Such test cases correspond to prediction of novel druggable target-disease relationships. Lastly, we selected the best performing machine learning model and predicted novel indications for a set of druggable genomes which currently have no approved indications, and we validated the quality of these predictions by comparing them to text mining results. A case study examining results for the highly pursued targets IL-12B and IL-23R was also conducted and reveals potential new indications uncovered by this approach.

## Results

### Overview and processing of Open Targets data

We downloaded association evidence information for all target-disease direct association pairs from the Open Targets Platform via API. Disease terms to be included were filtered to remove non-specific terms and we removed disease terms whose therapeutic areas belonged to measurement, phenotype, biological process, and/or cell proliferation disorders. The filtered data set then included 1,378,786 direct target-disease associations for 24,064 unique targets after this processing. Among these, there were 990 targets with at least one indication in clinical trials. OT associations for these target-indication pairs were used to build a working dataset for model evaluation, which consisted of 229,228 target-disease pairs. Another 23,074 targets and their indication associations were used as a prediction dataset for novel indication prediction. The working dataset was further split into training set and testing sets of 70% and 30% for model evaluation: the training set contained 159,249 target-disease pairs for 693 unique targets and the testing set contained 69,979 target-disease pairs for 297 unique targets.

**Fig. 2:**
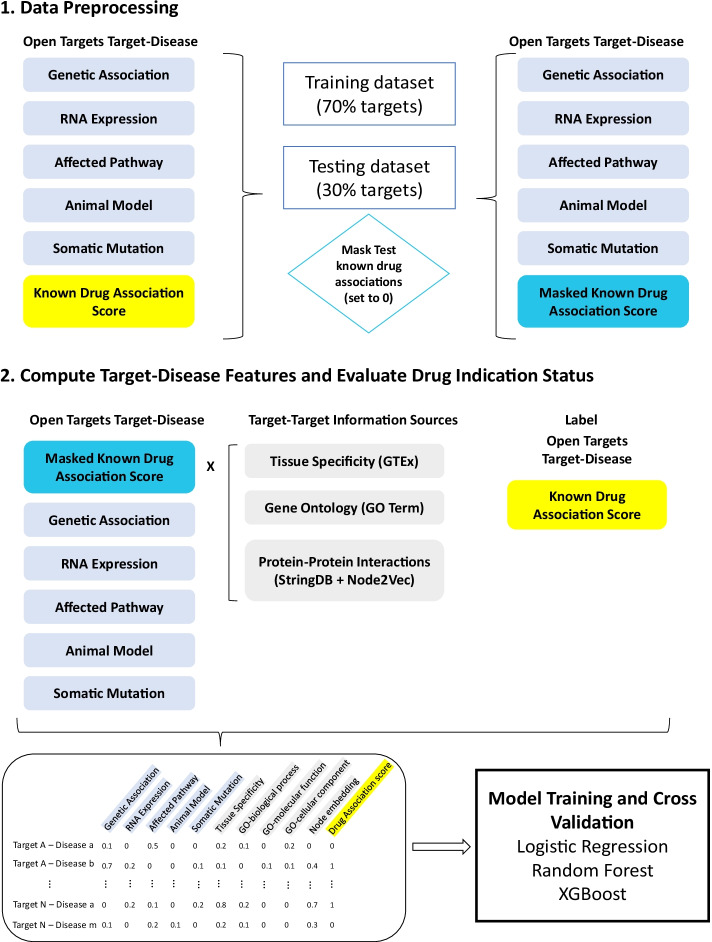
Workflow schematic for feature generation and therapeutic status prediction evaluation

Five available OT target-disease association features (genetic associations, somatic mutations, affected pathway, RNA expression and animal models) were used to predict known drug indication status for each target-disease pair by their association scores. For this prediction task, we used OT association scores for known drug status to define a binary label, with target-disease pairs with at least one drug in clinical trials receiving the label 1 and target-disease pairs with no known drugs in trial labelled 0 (Fig. 2).

Five new features were generated using the collaborative filtering-based approach. To compute these features, target-target similarity matrices were first generated from target co-expression similarity on tissues, semantic similarity from gene ontologies (Molecular Function (MF), Cellular Component (CC) and Biological Process (BP)), and node embedding similarity derived from protein–protein interaction networks. Then for each target, single new features by data source were computed from each target’s most similar 1000 genes and their known association scores of drug status in OT (Fig. 1c). To avoid contaminating our predictive feature with test case information, using cross-validation, before feature encoding, we masked known associations of drug status for validation test dataset target-indication pairs. This step was taken when calculating new features in order to avoid introducing ground truth before prediction. Training and test data sets then had 10 features, five of which were Open Targets Association scores (Genetic Association, RNA Expression, Affected Pathway, Animal Model, Somatic Mutation) and five newly computed features. The newly computed features were generated as the product of an array of target-target similarities for the 1000 most similar targets from each orthogonal data resource (GTex Tissue Specificity, GO-Biological Process, GO-Molecular Function, GO-Cellular Component, and StringDB Node Embedding) and the matrix of Open Targets target-disease Known Drug Association scores for these 1000 most similar targets. This calculation yielded a set of target-disease association scores for each data resource which is used as input for the target-disease indication status prediction task. The schematic input table with all features is shown in Fig. 2. All features arere continuous. Missing values were filled with 0, as missingness is interpretable as no evidence for interaction found between the target and indication. Data with 0 values in all features were removed for prediction tasks. Table 1 summarizes the number of samples in training, validation, and test datasets after removal of all-zero data rows.

**Table 1:** Number of data instances used for training and validation after removal of all-zero value rows

Set	Fold1	Fold2	Fold3	Fold4	Fold5
*Train*					
Positive	15,137	14,382	15,120	14,435	14,918
Negative	70,945	67,020	70,210	73,575	71,941
Total	86,082	81,402	85,330	88,010	86,859
*Validation*					
Positive	3369	4085	3404	4098	3561
Negative	18,132	20,313	18,424	15,344	16,194
Total	21,501	24,398	21,828	19,442	19,755

Held-out testing data comprised of 46,290 instances (7382 positive: 38,907 negative)

### Prediction performance evaluation by machine learning models

**Fig. 3:**
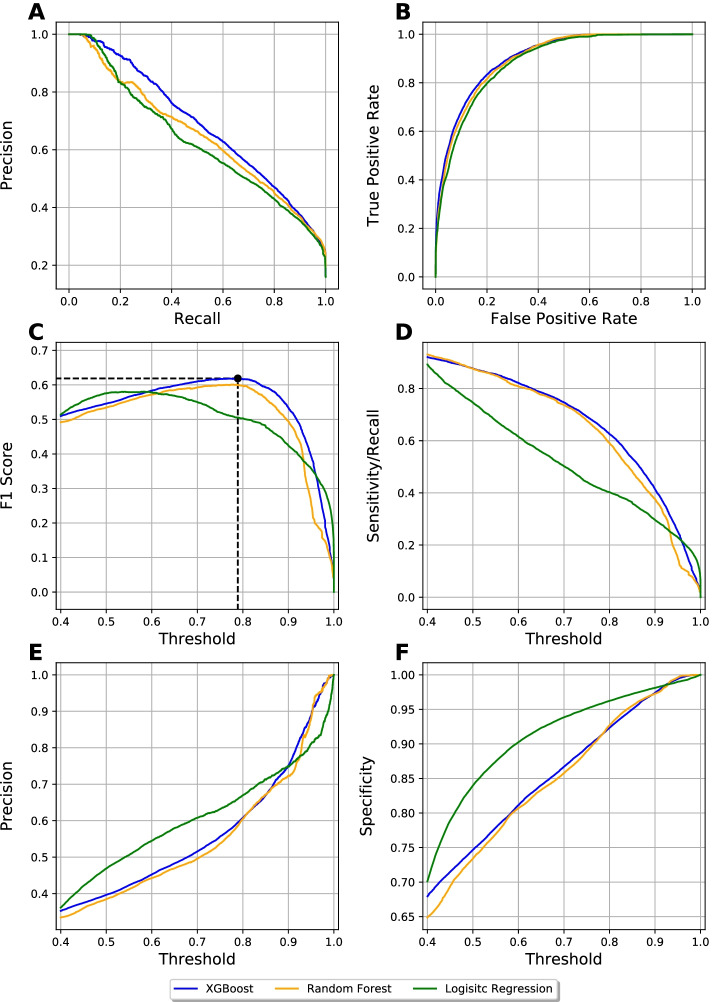
Known drug prediction performance in Testing set by XGBoost, Random Forest and Logistic Regression. Precision-Recall curve (**a**), Receiver operating characteristic curve (**b**), F1 score (**c**), Sensitivity (**d**), Precision (**e**) and Specificity (**f**)

We selected three machine learning models (logistic regression, random forest (RF) [18] and XGBoost [19] and performed fivefold cross validation with train-test split to tune the hyperparameters and evaluate performance of their classifications. From fivefold cross validation results, we found that XGBoost had the best performance with AUPR = 0.73 in the validation set, outperforming RF and logistic regression, as shown in Table 2. The model also performed well consistently with the testing set, with AUPR = 0.69, outperformed RF (AUPR = 0.65) and logistic regression (AUPR = 0.63) (Fig. 3a). Additionally, we observed XGBoost and RF got higher sensitivity and lower specificity compared to logistic regression. With the best cut-off derived from the maximum F_1_-score, which is 0.78, XGBoost model achieved a recall rate 0.75, precision 0.52 and F_1_ score 0.61 (Fig. 3b–d). However, with a common cut-off in traditional binary classifier, which is 0.5, the recall increased significantly to 0.88 while precision decreased to 0.40 (Fig. 3e, f). In a positive-unlabeled problem, the unlabeled data consists of both potential positive and negative data instances, but these all are considered as negative labels in training. Thus, it is not surprising to see a high false positive rate in prediction. According to benchmarking, we selected XGBoost for further predicting novel indications.

**Table 2:** Known drug status prediction (± standard deviation across 5 folds)

Method	LogReg	RF	XGB
*Train set*			
OT association evidence			
AUROC	0.7603 (± 0.0088)	0.8685 (± 0.0075)	0.8784 (± 0.0051)
AUPR	0.0685 (± 0.0025)	0.2074 (± 0.0093)	0.2072 (± 0.0103)
Computed features + OT association evidence			
AUROC	0.8867 (± 0.0027)	0.9262 (± 0.0019)	0.9406 (± 0.0018)
AUPR	0.6442 (± 0.0069)	0.7500 (± 0.0070)	0.7969 (± 0.0065)
*Validation set*			
OT association evidence			
AUROC	0.7625 (± 0.0357)	0.8143 (± 0.0314)	0.8076 (± 0.0335)
AUPR	0.0707 (± 0.0102)	0.0872 (± 0.0118)	0.0888 (± 0.0177)
Computed features + OT association evidence			
AUROC	0.8864 (± 0.0076)	0.9103 (± 0.0140)	0.9137 (± 0.0142)
AUPR	0.6452 (± 0.0226)	0.7092 (± 0.0459)	0.7264 (± 0.0457)

As a benchmark, we then evaluated the prediction accuracy using only the 5 association scores taken directly from Open Targets as well. The AUPR of XGBoost and Random Forest model in train set is around 0.2, and 0.1 in testing set (Table 2). That suggested training a prediction model with 5 association scores was underfitting. By taking newly computed features into consideration, prediction performance is significantly increased.

We also then explored the relationship between the continuous prediction score and drug status. From the testing set, every target-disease pair was compared with the highest stage of clinical trials achieved before for that target-indication pairing. With the target-disease pairs that have high prediction score, which is larger than 0.78, we found more than 70% approved indications, more than 65% indications in phase III and over 50% indications in phase I/II were identified successfully (Additional file 1: Figure 1).

To further investigate the prediction performance in a particular disease type, in testing set, we evaluated the AUPR for each disease respectively. Additionally, by grouping diseases into therapeutic areas, we understood in which therapeutic areas the predictive model performed well (Additional file 2: Figure 2). The predictive model had great performance in most therapeutic areas, especially in nutritional or metabolomic disease, diseases of the visual system, and nervous system disease. With more exploration on the data, we found the difference of accuracy was affected by the proportion of positive labels (known indications). Diseases with higher accuracy (AUPR > 0.6) have more than 30% known indications on average, while diseases with AUPR < 0.5 only have around 10% known indications (Additional file 5: Table 1), which is as expected since those unlabeled indications still have the opportunities to be selected in the future. In the same way, we also investigated the prediction performance in each target, where we reached a similar conclusion that the targets with better performance (AUPR > 0.6) have around 40% known indications on average and target with AUPR < 0.5 only have 6% known indications (Additional file 6: Table 2).

### Newly generated features demonstrate higher feature importance

**Fig. 4:**
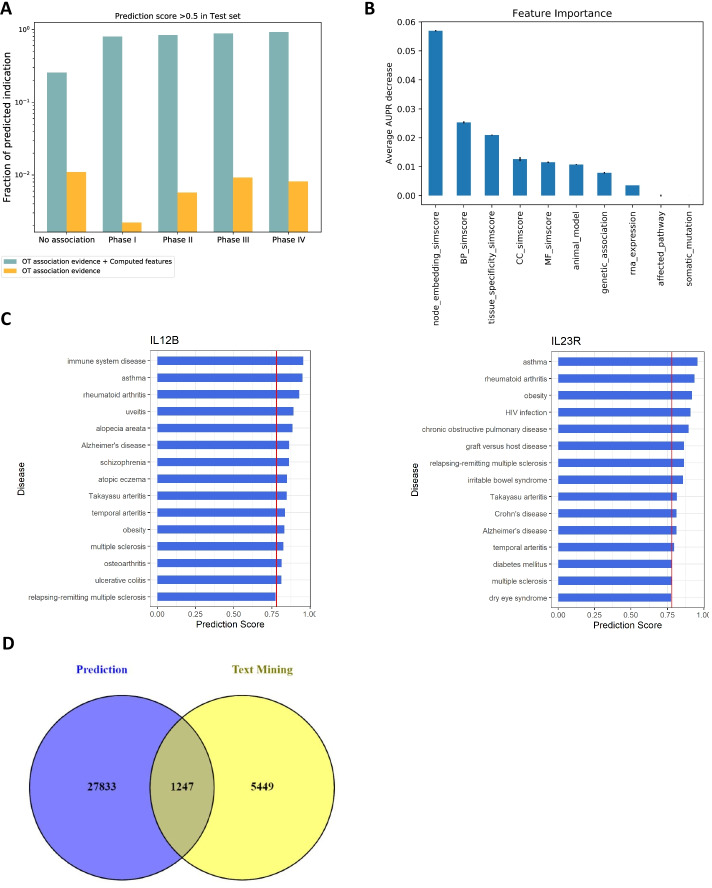
Newly computed features improve prediction accuracy. Prediction scores correspond to Testing set target-disease clinical trial stage (**a**). Feature importance scores indicate the feature types we generated strongly predict known drug therapeutic status (**b**). Target-disease arrays computed using target-target similarity reveal druggable target-disease pairs (**c**). Significant overlap between predicted indications and literature findings by text mining (**d**)

To compare relative importance of OT association evidence versus our newly created biological similarity features, feature importance was evaluated by permutation in the highest-performing XGBoost model. For each variable, we calculated the average decrease of AUPR by randomly shuffling variables. A feature is more important when the performance decreases more. When examining the feature importance scores obtained from model training, we identified network embedding information as the most important predictor for the task of identifying target-disease pair indication status (Fig. 4b). Interestingly, all computed features were assigned greater importance than association scores in this evaluation, indicating that our newly generated features contribute meaningful and relevant information for this task. In the interpretation of these results, how the known drug status is used for feature generation may be considered further.

A visualization of how computed features which embed function- and tissue-level target biological networks outperform association evidence for therapeutic status prediction is shown in Fig. 4a. The Y-axis in the figure is log-scaled. Using a prediction score of 0.5 as a cut-off, more than 90% of approved drugs, and more than 80% of drugs in phase I/II/III were spotted by including computed features in the model. These results further suggest that while association evidence alone is limited in its use for revealing therapeutic status, newly computed features integrating target-target similarity empower the prediction of these target-disease associations.

### Using XGBoost to predict novel indications on druggable genomes

Given the strong performance of machine learning models in our evaluation, we used XGBoost and predicted novel indications for a set of druggable genomes. The full list for the druggable genome was obtained from the study of Finan et al*.* [20], which consists of 4,463 genes. We compared the list with all the OT targets with approved drug status and found there are 2,858 druggable genes that currently do not have any approved indications. Similarly, there is also a critical need to explore indication expansion for approved targets for many other therapeutics to benefit patients with diseases which are not approved indications. Incorporating biological evidence via our encoding method with curated association evidence in OT offers us an opportunity for leveraging our approach to identify novel drug-target-indications groupings that revealed by these new features and machine learning methods. Since there is no gold standard to evaluate such novel predictions for indication expansion or drug repurposing potential, we utilized the scientific literature as an external source of validation by retrieving suggested drug targets from published articles and checking what proportion of these were predicted with our model. Specifically, we searched for literature hits of a gene or protein being mentioned as a (potential) therapeutic target in titles and abstracts on MEDLINE. We found a large number of text mining instances corresponding to 6,696 unique diseases that co-occurred with the 2,858 druggable genomes. There were 1,247 in common diseases between the predicted set and text mining hits, representing a highly significant proportion as assessed by hypergeometric test (p = 6.33e-113) (Fig. 4d; Additional file 7: Table 3). Though our prediction is based on the druggable genome, it is worth mention that the method is equally applicable to non-druggable classified genes, since this druggability is likely to change over the years as emerging technologies such as PROTAC [21], RNAi [22], and CRISPR/Cas9 [23] to maximize their potential in drug discovery.

#### A case study on novel target indication expansion

We further conducted a case study on highly pursued drug targets interleukin 12B (IL-12B) and interleukin 23 receptor (IL-23R) to evaluate the performance of our predictive method.

IL-12B is a common subunit of interleukin 12 (IL-12) and Interleukin 23 (IL-23) [24]. There are various drugs developed to targeting IL-12B to modulate IL-12 (Th1)/IL-23(Th17) pathways for treating autoinflammatory diseases in recent years [25, 26], such as Ustekinumab. Ustekinumab was approved for the treatment of moderate-to-severe Psoriasis (2009), Psoriatic arthritis (2013), moderate-to-severe Crohn's disease (2016) and Ulcerative colitis (2019)( https://www.drugs.com/history/stelara.html); further studies would elucidate its potential role as first-line therapy for other autoinflammatory diseases. There is strong interest within biopharma to expand the therapeutic base for Ustekinumab and other similar drugs.

We applied the XGBoost model on IL-12B target to prioritize its indications including our additional computed predictive features. When we investigated the ranked top 50 indications (Table 3), we have found that Ustekinumab’s approved indications, or indications which are under clinical trials for IL-12B intervention were highly ranked in the list (Fig. 4c; Additional file 8: Table 4). Overall, looking at the approved/in-clinical-trial indications in the ranking list, the AUROC is 0.90. Among the top ranked indications, Ulcerative Colitis, which is one of the approved indications for Ustekinumab, was ranked 14th in the predicted list. Other approved indications for Ustekinumab were also with high rankings among all the predicted indications: Psoriatic arthritis (23rd), Crohn’s disease (26th), and Psoriasis (31st). We further noticed that for several other top-ranked indications, such as systemic lupus erythematosus (20th), ankylosing spondylitis (21st), the drug was investigated in multiple phase 3 clinical trials, while rheumatoid arthritis (3rd) and multiple sclerosis (12th) were linked with several clinical trials in phase 1 or phase 2, according to Informa pharmaceutical pipeline database (https://citeline.informa.com/).

**Table 3:** Top predicted indications for Ustekinumab

Ranking	Disease	Disease_ID	Prediction_score
1	Immune system disease	EFO_0000540	0.95580226
2	Asthma	EFO_0000270	0.94961476
3	Rheumatoid arthritis	EFO_0000685	0.9285401
4	Uveitis	EFO_1001231	0.8917595
5	Alopecia areata	EFO_0004192	0.8835659
6	Alzheimer's disease	EFO_0000249	0.8625836
7	Schizophrenia	EFO_0000692	0.862216
8	Atopic eczema	EFO_0000274	0.84960294
9	Takayasu arteritis	EFO_1001857	0.8476705
10	Temporal arteritis	EFO_1001209	0.8364161
11	Obesity	EFO_0001073	0.8306895
12	Multiple sclerosis	EFO_0003885	0.82465583
13	Osteoarthritis	EFO_0002506	0.8140218
14	Ulcerative colitis	EFO_0000729	0.8110609
15	Relapsing–remitting multiple sclerosis	EFO_0003929	0.7750288
16	Behcet's syndrome	EFO_0003780	0.75113726
17	Diabetes mellitus	EFO_0000400	0.74368954
18	Juvenile idiopathic arthritis	EFO_0002609	0.7401242
19	Non-alcoholic steatohepatitis	EFO_1001249	0.7374891
20	Systemic lupus erythematosus	EFO_0002690	0.73320544
21	Ankylosing spondylitis	EFO_0003898	0.7330974
22	Graft versus host disease	MONDO_0013730	0.72582567
23	Psoriatic arthritis	EFO_0003778	0.71739846
24	Alcohol dependence	EFO_0003829	0.71080697
25	Dermatitis	MONDO_0002406	0.7098975
26	Crohn's disease	EFO_0000384	0.6959177
27	HIV-1 infection	EFO_0000180	0.6868525
28	Periodontitis	EFO_0000649	0.6834563
29	Tuberculosis	Orphanet_3389	0.67681295
30	Post-traumatic stress disorder	EFO_0001358	0.6721301
31	Psoriasis	EFO_0000676	0.67159593
32	Viral disease	EFO_0000763	0.67042077
33	Cystic fibrosis	Orphanet_586	0.6682126
34	Abdominal Aortic Aneurysm	EFO_0004214	0.653386
35	Psoriasis vulgaris	EFO_1001494	0.63942015

We also performed the predication of novel indications for interleukin 23 receptor (IL-23R) (Fig. 4c; Additional file 9: Table 5), which interacts with IL23. Although there are currently no approved indications for drugs targeting IL-23R, the potential indications were expected to be similar as Ustekinumab. In our IL-23R top ranked list, we observed that Ustekinumab’s approved indications were among the top predicted indications on this drug target, including Crohn’s disease (10th) and Psoriasis (27th), indicating their high potential for IL-23R. Other novel indications revealed by this method may also be worth further investigations. The full list of our predicted indications for IL-12B and IL-23R can be found in the labelled supplementary files. The results of these cases study queries provide additional validation of the utility and power of our approach.

## Discussion

In this study, we present the development and application of a systematic indication prioritization approach which generates new target-disease features to supplement OT association evidence and evaluate the performance of these features for therapeutic status prediction in different machine learning models. Our current study demonstrates the benefit of combining OT association evidence network data with independent sources of biological knowledge base information in order to examine target-disease predictions with increased confidence in their validity. To our knowledge, our work provides the first proof-of-concept results for the complementarity of OT features with new in-silico target-disease data from tissue similarity, interconnectivity and semantic similarity. Our results confirm that our approach can play a key role in uncovering real insights in target-disease discovery work, expanding on the base OT network of targets and their disease associations. Combining promising disease association profiles, drug targets could be tested in new therapeutic areas where compelling evidence exists. In our analysis, we found that a threshold of 0.78 gives the highest F_1_-score, with recall 0.65 and precision 0.60. This suggests that a prediction score of 0.78 from XGBoost could be used as a stringent cut-off when evaluating potential new target-indication hypotheses, and that is also used in our prediction of novel indications. Meanwhile, since in the positive-unlabeled problem, recall should be weighted more than precision, we investigated with F_1.5_-score, as well. We found that a threshold of 0.62 gives the highest F_1.5_-score (Additional file 3: Fig. 3), with recall 0.81 and precision 0.47. Therefore, a prediction score of 0.62 from prediction model could be used as a moderate confidence cut-off. Cohen’s Kappa and Matthew’s correlations coefficient (MCC) with the two different thresholds in testing set by XGBoost and MCC were also calculated (Additional file 4: Figure 4A) and Cohen’s Kappa with different thresholds in testing set plotted for the three predictive models (Additional file 4: Figure 4B).

On the interpretation of the results of this analysis, how the known drug status is used for feature generation may be considered further. Test case target-disease pairs were assigned known drug associations of 0 prior to embedding to cancel this information during training. Under these circumstances, good model performance for recovery of true indication status is achieved either if OT target-disease association scores are themselves predictive of indication status or when target-target biological relatedness features correctly represent links between the target-disease pairs with hidden labels and other(s) target-disease pairings which are correctly labelled and have a known drug.

There are also aspects on our method which could be further improved. First, our method leveraged 10 features for the machine learning model training and prediction, 5 from OT and 5 from newly generated features. Therefore, it is important to note that the target-indication discovery by our approach can only be as accurate as the current features can represent the underlying biological process for each disease. To further improve the prediction of target-disease associations, other reliable features may need to be taken into consideration. For example, CRISPR-KO data in recent OT updates [2] could be further added in our approach. An advantage of the approach is that its formulation provides a straightforward framework for further data source integration. Additional target-target similarity matrices from other data sources may be readily adapted for new feature generation, such as gene co-expression in cell types, and protein structure similarities. Second, our method took target-target similarities into account and generated new features representing target-disease association. While we found diseases in Open Targets form a hierarchical structure, it would also be worthwhile in future work to explore the disease hierarchy for further improving prediction in the future. Disease-disease similarities could be investigated from disease biology and interactions and adapted to generate new features. Third, compared to the single machine learning model selected in our approach, ensembles of diverse machine learning models can generally outperform any individual model [27], e.g. using weighted average of the predictions from the different models, and future work might be undertaken to evaluate ensemble methods. In a recent Novartis-MIT data science and artificial intelligence challenge on predicting drug approvals, the winner was an ensemble consisting of two XGBoost models and one Bayesian logistic regression (BLR) model [28]. These results inform us with ways to design workflows to further improve method performance in our future studies.

It should also be noted that as with any computational approach, false positive and false negative results are unavoidable and should be expected, especially when addressing positive-unlabeled problems. The unlabeled data consists of both potential positive and negative data, so it’s not surprising that false positives were observed when testing prediction performance.

Each target-disease pair merely represents a hypothesis that serves as a starting point for drug discovery scientists looking to begin a new research program. These hypotheses still require careful evaluation, prioritization, and experimental validation.

## Conclusions

In summary, by utilizing the target-disease evidence from Open Targets platform with the integration of novel generated features, we have been able to generate various indication pair combinations, which form the basis for development hypotheses for potential drug discovery programs, and this approach can be generalized in a straightforward fashion to include other drug target classes and information resources. These methods also offer great potential for helping other investigators to develop better ways to utilize the fast-growing data in the Open Targets platform to reposition drugs for unmet medical needs, and the work we describe here could help identify possible new uses of existing drugs to be investigated further. Prospective clinical trials would be required to provide the necessary evidence to have such new uses approved by regulatory agencies.

## Methods

### Open Targets data processing

The target-disease association data was collected from Open Targets Platform, which was released in April 2021. It provides several types of score to represent the association between a gene and a disease based on evidence from 19 data sources, including genetic associations, somatic mutations, drugs, affected pathway, RNA expression, text mining and animal models. All the association scores range from 0 to 1. For each type of association score, it collects the evidence from one or multiple data sources. For example, genetic association is generated based on the clinical significance assessment from the European Variation Archive (https://www.ebi.ac.uk/eva/), GWAS Catalog [29], Gene2Phenotype [30], PheWAS catalogue [31] etc. And the drug association is calculated according to the evidence from ChEMBL [32]. Diseases in the Open Targets Platform are labeled based on Experimental Factor Ontology (EFO) [33], and classified into different therapeutic areas according to the relationships in EFO hierarchy. There are both direct and indirect associations in the database, we only kept direct associations to avoid artificially duplicated information.

We selected five association scores as feature spaces to predict novel target-indication pairs, including genetic associations, somatic mutations, affected pathway, RNA expression and animal models. The drug score in Open Targets Platform was used as labels in the prediction, which was collected from ChEMBL and generated based on clinical trials. A gene was labeled as drug target of a certain disease when the drug score is larger than 0. To focus on the non-oncology disorder, we further removed the diseases whose therapeutic areas belong to measurement, phenotype, biological process, and cell proliferation disorder.

### Generation of new features

In order to derive the target-target similarity matrix based on tissue specificity, the RNA-seq data were downloaded from GTEx v8 [34] where the samples were obtained from 54 tissues in 30 tissue groups. We removed lowly expressed genes and only kept the genes with at least non-zero count in one tissue. Limma package [35] was used to estimate tissue specificity by comparing samples in one tissue with samples in all other tissues from different tissue groups, we built the linear model by taking age and gender as covariates. Then we estimated gene similarity by computing cosine similarity on the tissue specificity matrix.

To obtain semantic similarity matrix among gene pairs, we estimated gene similarities through gene ontology (GO). Gene ontology comprises of three ontologies: biological process, molecular function, and cellular component. We used GOSemSim package [36] to compute the sematic similarity of genes based on the annotation statistics of their common ancestor terms. Specifically the Wang method [37] was employed to estimate GO sematic similarity, which utilized the topology of GO graph structure. It estimated the semantics of one gene from the aggregated contributions of all terms in the sub graph, which includes the gene and all of its ancestor terms in GO graph. We used this graph-based approach to estimate semantic similarity of genes, and generated three matrices from the gene ontologies respectively.

Protein to protein interaction network was downloaded from STRING database [16] to calculate node embedding similarity matrix for gene pairs. The STRING database is one of the most comprehensive protein to protein interaction network with predicted and known interactions. Each edge is given a weight to identify the degree of confidence. In order to generate a reliable, high-trust level network reference for our approach, we kept interactions with confidence score greater than 0.5 defined by STRING. There are many analysis approaches could be used to estimate node similarity in a network, such as random walk [38], network propagation [39], etc. In our approach, we used node2vec [17], a deep learning model which performs random walks through the network by starting at a random node and following a series of steps to random neighbors. Each of these random walks forms a sentence that can be fed to word2vec [40] to generate the embedding for each node. Compared to other algorithms, node2vec detects homophily and structural similarities using depth and breadth-first search, generate node embeddings that can be expanded to predictive models for deeper investigation. The gene similarities then can be systematically computed with each node embeddings by cosine similarity.

New features for each gene were then generated based on a prior known target-disease matrix (from OT target-disease known drug status) and gene similarity matrix. For example, to calculate new features for gene A, the top 1000 most similar genes were selected for gene A based on each type of gene similarity matrix, and used for generating new features later. The associations of these 1000 genes to disease could be derived from prior known target-disease indication matrix, then new features of gene A with diseases was weighted average associations from top 1000 most similar genes, where weights were from gene similarity matrix (Fig. 1c). By this approach, each target receives a new disease-association score for each added data source which is computed to yield association scores with all k ϵ K disease terms for each Target_j_ through the following equation:

$$Disease \;Associations \left( {Target_{j} ,Disease_{k} } \right) = \frac{{\mathop \sum \nolimits_{i = 1}^{1000} Similarity\left( {Target_{j} ,{ }Target_{i} } \right)* OT\; Known \;Drug \;Associaton \left( {Target_{i} ,{ }Disease_{k} } \right) }}{{\mathop \sum \nolimits_{i = 1}^{1000} \left| {Similarity\left( {Target_{j} ,{ }Target_{i} } \right)} \right|}}$$

### Machine learning model training and evaluation

The genes which have at least one indication in clinical trials and their associations were selected to generate a working dataset for training and evaluating machine learning models. Other genes that don’t get approved in any indication before were collected as prediction dataset, which would be used to discover novel indications with well-trained model.

The working dataset was further split into training set and testing set, which contains 70% and 30% genes and their corresponding associations respectively. With the training set, we performed fivefold cross validation to tune hyperparameters and evaluate performance of three classifiers, including logistic regression, random forest [18] and XGBoost [19]. We used grid search to tune maximum depth of a tree, minimum sum of instance weight in a child, subsample ratio of the training instances, the subsample ratio of columns when constructing a tree, weight of L2 regularization term, and learning rate in XGBoost. After tuning, the hyperparameters used in XGBoost were min_child_weight = 10, colsample_bytree = 0.6, subsample = 0.6, max_depth = 8, reg_lambda = 10, eta = 0.1, while all the other hyperparameters were used with default values. We tuned the number of trees, the maximum depth of a tree, the number of samples to train each tree, and the number of features to train each tree in Random Forest. After tuning, the hyperparameters used in Random forest were n_estimators = 500, max_depth = 8, max_samples = 0.6, max_features = 0.6 and all the other hyperparameters were used with default values.

Since our dataset is extremely imbalanced, unlabeled data is 8 times as many as positive data. We computed a class weight to adjust loss function when training models for solving the issue of imbalance. Meanwhile, when addressing positive-unlabeled problem, overfitting is a common issue with traditional classifiers, since the unlabeled data is considered as negative, but it could consist of both positive and negative data in fact. We can prevent overfitting by using bagging when building models, which is randomly resampling from the original dataset with replacement and making prediction with majority votes. While XGBoost and random forest have bagging built-in, we only applied bagging with 100 iterations in logistic regression, the final prediction was derived from average score from 100 iterations.

In the cross validation, we took area under precision-recall curve (AUPR) as evaluation metrics. According to the evaluation scores in benchmarking, XGBoost was selected with a better prediction performance, and was further applied to testing set for evaluation. XGBoost consistently showed better performances in both cross validation and testing set, so it was used to predict novel indications with prediction dataset and further validated by text mining.

### Text mining

The text mining tool based on natural language processing and linguistic analytics I2E from Linguamatics were utilized to evaluate the predictions from XGBoost model. An I2E query on “Genes_known_as_targets” was built to extracts target and indication hits from titles and abstracts stored in the MEDLINE database. We defined “Genes” as druggable genes which currently have no approved indications. This query looks for these genes which are explicitly mentioned in the literature as potential targets of a disease, where “Gene” stands for the druggable genes and “Target” is a concept describing a therapeutic target. MeSH terms were used for diseases in the text mining hits so that we can use corresponding MeSH IDs to map across Open Target EFO terms. Results were retrieved and processed in an excel table. A hypergeometric test was performed to assess the significance of the overlap between predictions and text mining hits using the total number of druggable genes as the universe size.

## Supplementary Information

**Additional file 1**. **Supplement Figure 1.** Prediction score and clinical trial stage.

**Additional file 2**. **Supplement Figure 2.** Model performance by therapeutic area.

**Additional file 3**. **Supplement Figure 3.** Prediction score cutoff by F1.5 score.

**Additional file 4**. **Supplement Figure 4.** Cohen’s Kappa and Matthew’s correlations coefficient (MCC) with the two different thresholds in testing set by XGBoost (A). MCC and Cohen’s Kappa with different thresholds in testing set by 3 predictive models (B).

**Additional file 5**. **Supplement Table 1.** Disease Level Performance.

**Additional file 6**. **Supplement Table 2.** Target Level Performance.

**Additional file 7**. **Supplement Table 3.** OT Novel Indication Predictions Druggable Genome.

**Additional file 8**. **Supplement Table 4.** IL12B Ranking List.

**Additional file 9**. **Supplement Table 5.** IL23R Ranking List.

## Data Availability

Datasets used and analyzed during the current study are publicly available through the Open Targets platform (https://www.opentargets.org/).
