# Supplementary material for: Empowering the discovery of novel target-disease associations via machine learning approaches in the open targets platform
Source: BMC Bioinformatics. 2022 Jun 16;23:232. doi: 10.1186/s12859-022-04753-4 (PMC9202116; doi:10.1186/s12859-022-04753-4)
Supplement: Supplementary file 1 — Additional file 1. Supplement Figure 1. Prediction score and clinical trial stage. [file 12859_2022_4753_MOESM1_ESM.pdf]

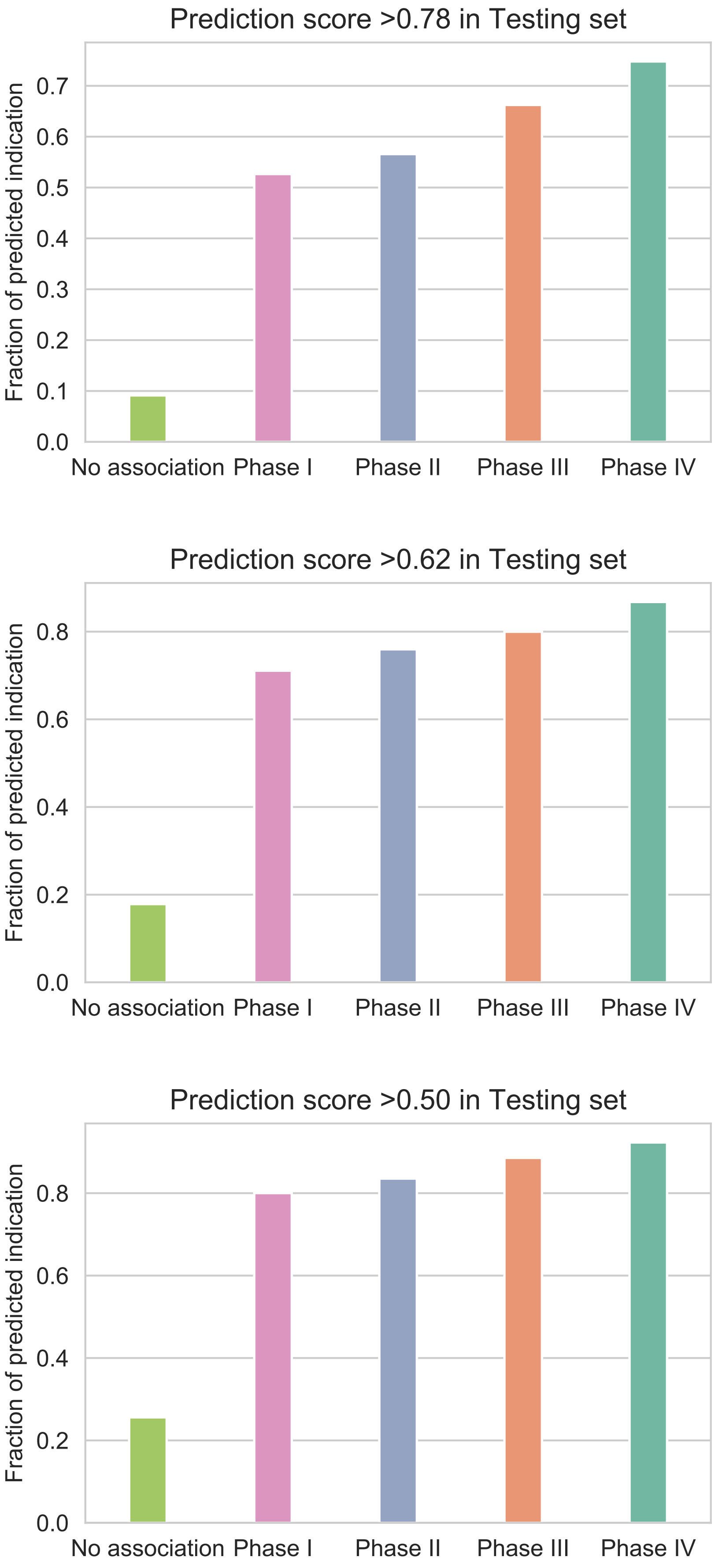
Prediction score >0.78 in Testing set
Fraction of predicted indication
0.0
0.1
0.2
0.3
0.4
0.5
0.6
0.7
No association
Phase I
Phase II
Phase III
Phase IV
Prediction score >0.62 in Testing set
Fraction of predicted indication
0.0
0.2
0.4
0.6
0.8
No association
Phase I
Phase II
Phase III
Phase IV
Prediction score >0.50 in Testing set
Fraction of predicted indication
0.0
0.2
0.4
0.6
0.8
No association
Phase I
Phase II
Phase III
Phase IV
