# Supplementary figures and images for: Empowering the discovery of novel target-disease associations via machine learning approaches in the open targets platform
Source: BMC Bioinformatics. 2022 Jun 16;23:232. doi: 10.1186/s12859-022-04753-4 (PMC9202116; doi:10.1186/s12859-022-04753-4)

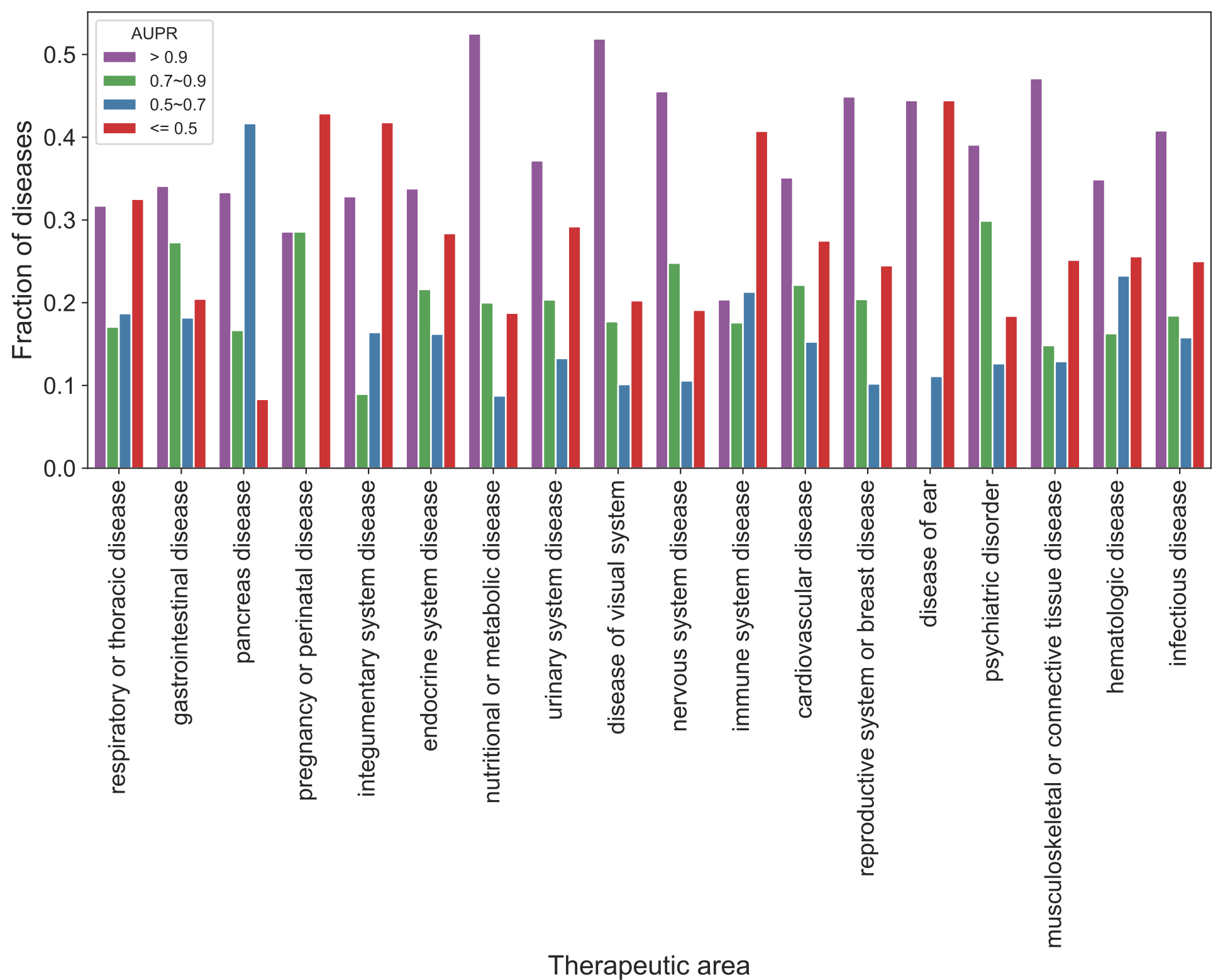

Supplement: Supplementary file 2 — Additional file 2. Supplement Figure 2. Model performance by therapeutic area. [file 12859_2022_4753_MOESM2_ESM.pdf]

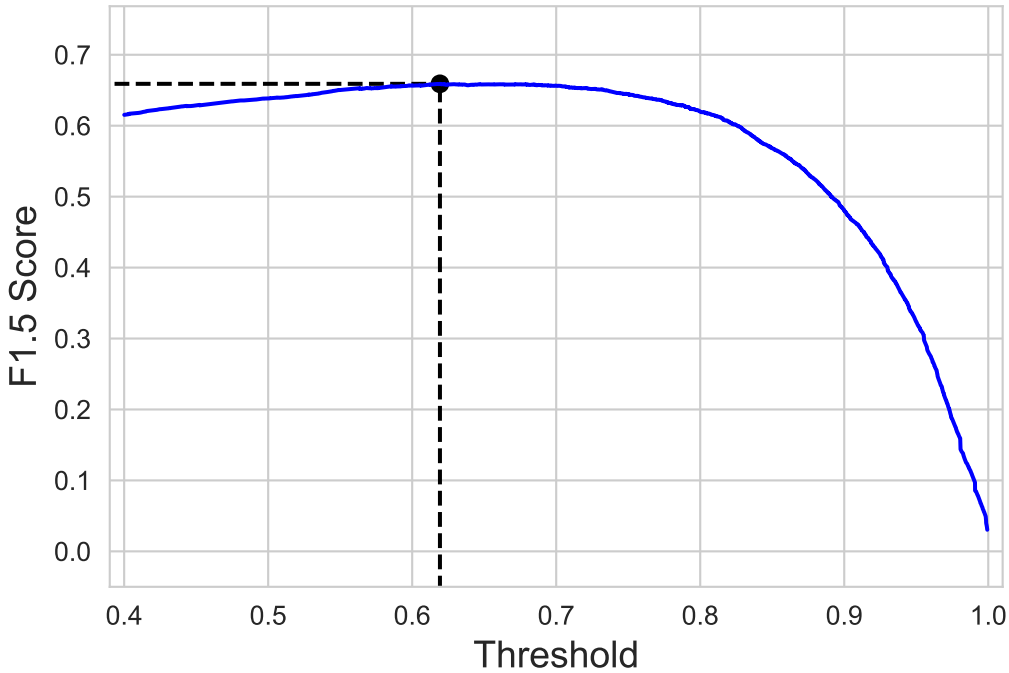

Supplement: Supplementary file 3 — Additional file 3. Supplement Figure 3. Prediction score cutoff by F1.5 score. [file 12859_2022_4753_MOESM3_ESM.pdf]
