# Supplementary material for: Empowering the discovery of novel target-disease associations via machine learning approaches in the open targets platform
Source: BMC Bioinformatics. 2022 Jun 16;23:232. doi: 10.1186/s12859-022-04753-4 (PMC9202116; doi:10.1186/s12859-022-04753-4)

A

	Cohen's Kappa	MCC
Cut-off = 0.78 (highest F1 score)	0.543	0.543
Cut-off = 0.62 (highest F1.5 score)	0.487	0.517

B

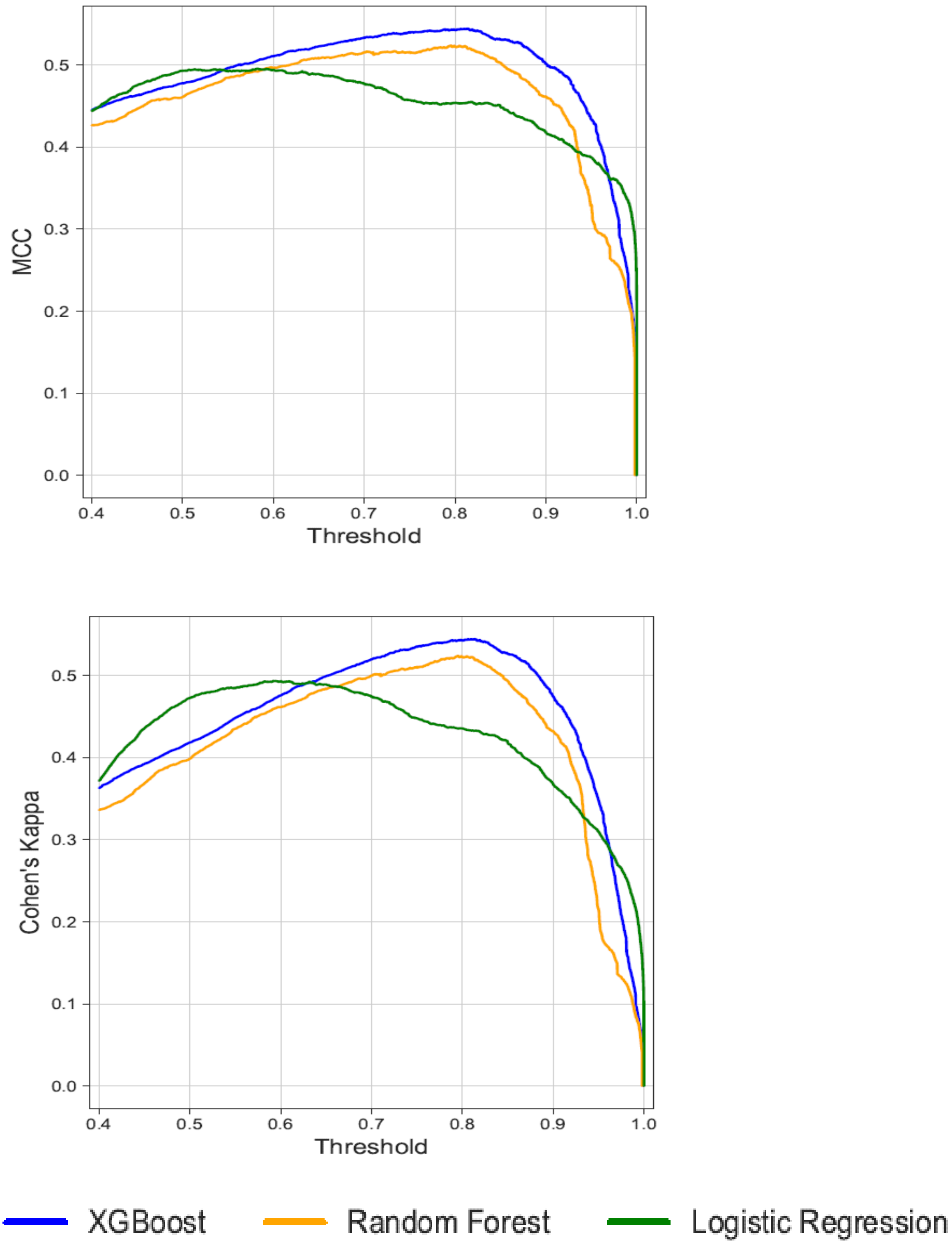

Supplement: Supplementary file 4 — Additional file 4. Supplement Figure 4. Cohen’s Kappa and Matthew’s correlations coefficient (MCC) with the two different thresholds in testing set by XGBoost (A). MCC and Cohen’s Kappa with different thresholds in testing set by 3 predictive models (B). [file 12859_2022_4753_MOESM4_ESM.pdf]
